# Sustainable Control of Water-Related Infectious Diseases: A Review and Proposal for Interdisciplinary Health-Based Systems Research

**DOI:** 10.1289/ehp.0800423

**Published:** 2009-04-17

**Authors:** Stuart Batterman, Joseph Eisenberg, Rebecca Hardin, Margaret E. Kruk, Maria Carmen Lemos, Anna M. Michalak, Bhramar Mukherjee, Elisha Renne, Howard Stein, Cristy Watkins, Mark L. Wilson

**Affiliations:** 1 Department of Environmental Health Sciences; 2 Department of Epidemiology; 3 School of Natural Resources and the Environment; 4 Department of Health Management and Policy; 5 Department of Civil and Environmental Engineering; 6 Department of Biostatistics; 7 Department of Environmental Engineering; 8 Center for African American Studies and; 9 School of Natural Resources and Environment, University of Michigan, Ann Arbor, Michigan, USA

**Keywords:** infectious disease, interdisciplinary, malaria, research, systems approach, water

## Abstract

**Objective:**

Even when initially successful, many interventions aimed at reducing the toll of water-related infectious disease have not been sustainable over longer periods of time. Here we review historical practices in water-related infectious disease research and propose an interdisciplinary public health oriented systems approach to research and intervention design.

**Data sources:**

On the basis of the literature and the authors’ experiences, we summarize contributions from key disciplines and identify common problems and trends. Practices in developing countries, where the disease burden is the most severe, are emphasized.

**Data extraction:**

We define waterborne and water-associated vectorborne diseases and identify disciplinary themes and conceptual needs by drawing from ecologic, anthropologic, engineering, political/economic, and public health fields. A case study examines one of the classes of water-related infectious disease.

**Data synthesis:**

The limited success in designing sustainable interventions is attributable to factors that include the complexity and interactions among the social, ecologic, engineering, political/economic, and public health domains; incomplete data; a lack of relevant indicators; and most important, an inadequate understanding of the proximal and distal factors that cause water-related infectious disease. Fundamental change is needed for research on water-related infectious diseases, and we advocate a systems approach framework using an ongoing evidence-based health outcomes focus with an extended time horizon. The examples and case study in the review show many opportunities for interdisciplinary collaborations, data fusion techniques, and other advances.

**Conclusions:**

The proposed framework will facilitate research by addressing the complexity and divergent scales of problems and by engaging scientists in the disciplines needed to tackle these difficult problems. Such research can enhance the prevention and control of water-related infectious diseases in a manner that is sustainable and focused on public health outcomes.

Why have interventions against infectious diseases often proven to be less successful than anticipated? Even when initially successful, many are not sustainable over longer periods of time. During the past 30 or 40 years, we have observed many and diverse examples where infectious disease reduction efforts have failed to meet expectations, with diseases reemerging to preintervention levels or worse. Dengue fever, for example, was eliminated from the Americas for many years only to reemerge with a more virulent form of the disease, including dengue hemorrhagic fever. Schistosomiasis and malaria both have shown that they can quickly reemerge after intervention efforts are loosened. Waterborne zoonotic agents such as *Escherichia coli* O157:H7, *Campylobacter jejuni*, and *Cryptosporidium parvum* have emerged in recent years (Cotruvo et al. 2004). Many other water-associated human pathogens, including *Vibrio cholerae* O139, hepatitis viruses, cyclospora, microsporidia, *Yersinia enterocolitica*, and environmental bacteria (e.g., *Legionella pneumophila*), have been associated with waterborne illnesses over the past few decades (Sharma et al. 2003). Thus, sustainability has become an important criterion for gauging the success of disease reduction efforts.

The nature and impact of water-related infectious diseases are mediated by both ecologic and socioeconomic processes [Eisenberg et al. 2007; United Nations Environment Programme (UNEP) 2007]. Although the complexity of these processes is being increasingly realized, current research and management approaches include only a subset of the considerations and interdisciplinary exchanges needed to approach and realize sustainable solutions. These issues represent major gaps that cannot be resolved by simply boosting funding for water supply and sanitation facilities. The need for new approaches to address the challenges of water-related infectious disease research motivates this review and assessment. In particular, we argue that one problem with interventions to reduce infectious disease incidence and emergence, particularly those that are water-associated, is that efforts are generally directed against proximal causes of infection transmission, paying less (and often insufficient) attention to the more distal causal factors. This proximal focus comes from an individual-based approach to etiology and epidemiology that emphasizes the immediate and short-term risk factors. We suggest that incorporating more distal processes into analyses and designs of interventions will result in more sustainable interventions. This new approach requires both systems-level thinking and an interdisciplinary approach to research and intervention design.

Here we review the different disciplinary approaches to infectious disease research and interventions and argue for an expanded interdisciplinary approach. We focus on water-related diseases (such as waterborne and vector-borne) and their more distal causes, which involve both social and ecologic processes. We summarize traditional and recent approaches, identify the main disciplinary themes, and discuss their strengths and weaknesses. We then propose an interdisciplinary, public health–oriented systems approach to research aimed at providing a comprehensive means to prioritize water-related health outcomes using evidence-based interventions (Ezzati et al. 2005). Finally, the suggested approach is illustrated using a case study that focuses on diseases associated with water and sanitation management practices in developing countries where the disease burden is the most severe.

## Background and motivation

Water- and disease-related issues are major roadblocks to sustainable development. As noted by Toepfer (2004), disease statistics are stark and tragic: 80% of illness and death in the developing world is water-related; half of the world’s hospital beds are occupied by people with water-related diseases; diarrhea and malaria are by far the largest causes of mortality in children < 5 years of age (34%) in Africa; and the number of deaths from water-related disease approaches 5 million annually, most of them children. These deaths, most of which are preventable, largely occur among the estimated 1.2 billion people worldwide without access to safe and reliable drinking water and the 2.5 billion without access to sanitation services. Despite ongoing efforts, the 2002 Millennium Development Goal of halving the population without clean water or sanitation by 2015 is unlikely to be achieved (United Nations 2008).

Many stressors affect water hygiene and sanitation. These include *a*) population growth, urbanization, and increasing population density that increases vulnerability to waterborne diseases; *b*) growing water demand by cities, industry, and agriculture, often coupled with limited opportunities for reservoir or aquifer development; *c*) climate variability and change that together erode food production capacity, diminish water availability and water quality, and increase flooding and drought due to inadequate drainage and storage; and *d*) “advancements” associated with development, such as dams, roads, deforestation, and agricultural irrigation, that lead to increased prevalence of water-associated disease (Patz et al. 2004). These factors can interact in ways that adversely affect water quantity, quality, sanitation, and health.

**Figure 1:**
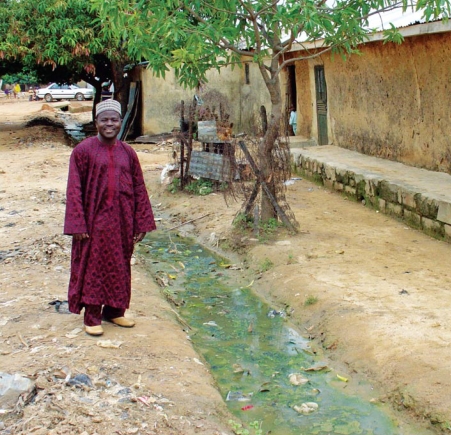
A drainage canal constructed by local community efforts in Zaria City, Nigeria, a malaria region. Dr. Samaila U. Dakyes, of the Department of Industrial Design, Ahmadu Bello University–Zaria, is a local chief who has organized people in his community to maintain sewers and waste control. Such efforts can improve conditions, but the stagnant water shown in the photo indicates the need for additional work and infrastructure. Photo by S. Batterman.

We group water-related infectious diseases into two categories. Waterborne infectious diseases, such as diarrhea, are linked to poor sanitation, inadequate hygiene, ingestion of and contact with unsafe water, and lack of access to adequate amounts of safe water [World Health Organization (WHO) 2008]. Water-associated vector-borne diseases, such as malaria and dengue fever, require water to propagate insect vectors (e.g., mosquitoes, black flies) that transmit pathogenic microbes when taking a blood meal from a human (WHO 2008). Another kind of water-associated disease, schistosomiasis, is caused by a worm or blood fluke whose life cycle involves particular aquatic snails and human contact with infected water. Habitat requirements of such insect and snail vectors are species-specific and can include large and small water bodies and channels (e.g., lakes, lagoons, rivers, ditches, culverts, sewers), poorly drained soils, and containers (e.g., pots, tires, leaves, tree stumps) (Figure 1). Many water-related infectious diseases have been referred to as the “neglected diseases of neglected populations,” because they receive little attention and disproportionately affect poor people in developing nations (Ehrenberg and Ault 2005).

## Sustainability and research

We adopt a definition of sustainable water use as the “use of water that supports the ability of human society to endure and flourish into the indefinite future without undermining the integrity of the hydrologic cycle of the ecologic systems that depend on it” (Gleick et al. 1995). This broad definition is necessary to appropriately address the more distal factors associated with disease burden, and it suggests a research agenda for sustainable water management that represents a profound change from focusing on specific human uses—for example, water treatment plants, wells, and sewage systems—to considering a more holistic system that encompasses both human and ecologic systems and infrastructure components. Furthermore, in this context, sustainability must be approached using interdisciplinary tools that span social, physical, and ecologic sciences, including health sciences. In practice, indicators of sustainability are context-or problem-specific, and programs that seek to advance sustainability must be flexible. To complete a cycle of planning, analysis, intervention, and evaluation, time frames must span one or preferably two decades, appropriate for economic discounting and forecasting, climate and other ecologic changes, and socio-cultural trends. As discussed below, many disciplines have contributed to an understanding of water-related disease. We present these disciplinary approaches, which help to motivate the need to move toward more integrated interdisciplinary approaches.

## Approaches to Water-Related Infectious Disease Research

As early as the Hippocratic writings, scholars recognized the connection between food, water, and the environment, along with the direct and changeable influence of human behavior on disease prevalence (Franco and Williams 2000). The Egyptians, Romans, Greeks, and other ancients had perfected many water supply, hygiene, and sanitation practices (el Gamili et al. 2001; Koutsoyiannis et al. 2008). In modern times, John Snow (the “father of epidemiology”) and Henry Whitehead pioneered the understanding of the causes of water-related disease transmission (Newsom 2006). Many disciplines have significant interests in water management and disease control, including those concerned with natural and physical processes (e.g., ecology and engineering), human dimensions (sociocultural and economic/political); and health outcomes (e.g., biology, ecology, epidemiology, parasitology). Such disciplines provide complementary frames that together can provide a comprehensive approach to understanding water-related infectious diseases. The following section highlights key themes from critical disciplines.

### Ecologic approaches

Water-related infectious disease research has long used an ecologic perspective to understand how best to control transmission (Eisenberg et al. 2007). Ecologic approaches to water-related diseases have drawn from biology, epidemiology, and genetics, among other fields, to focus on environmental determinants of disease via natural and increasingly anthropogenic changes to the physical environment, which frequently result from shifts in (human) population, consumption, and technologic growth (UNEP 2007). Often vectors, stressors, and disease can be mechanistically linked to these changes from micro (e.g., ponds, rivers, wells) to global scales (e.g., effects of climate change on water flow patterns and biomes).

Disease and microbial ecology research has identified multiple modes of transmission for both vectorborne and waterborne pathogens that depend on environmental, climatic, infrastructural, and sociocultural conditions (Wright et al. 2004). Many pathogens move about the environment via human feces (Curtis et al. 2000), and both humans and animals can act as hosts. Exposure to fecal pathogens occurs in both private (e.g., domestic living spaces, private yards, and fields) and public spaces (e.g., workplaces, transportation hubs, markets; Kosek et al. 2003) and is most often linked to poverty, poor education, and underdevelopment. Knowledge of the interactions between bacterial, viral, and parasitic enteric pathogens and the microbial system of the human gut can shed light on population differences in susceptibility (Mathew et al. 1991), whereas vector ecology can inform the design of interventions—for example, mosquito behavioral flexibility (Huang et al. 2006), flight range, and spatial distribution (Pope et al. 2005; Sirot et al. 2008). Disease ecology, however, cannot be seen as independent of social behavior and political economy.

Motivated by unsuitable and poorly targeted vector control programs, as well as diseases that are drug-resistant or for which there are no vaccines, the WHO has promoted integrated vector management (IVM) (WHO 2004), an ecologic approach that includes both vector ecology (promoting minimal use of insecticides) and public health initiatives (including community dialogue and participation). The need for increased community education, management integration, monitoring, and evaluation in such programs has been recognized (Mukabana et al. 2006; Townson et al. 2005; Van den Berg et al. 2007).

### Engineering approaches

Engineering approaches offer the science and technology for the design of safe (and adequate) water, sanitation, hygiene, and drainage systems, as well as the forecasting, operations, and management skills needed to maintain and optimize systems for a suitably long period. For millennia, scientific principles have been used to engineer both the flow and quality of water for drinking, irrigation, recreation, and other purposes; to handle solid and liquid wastes; to drain (and, more recently, restore) wetlands; and to provide flood control. Water-related infrastructure, including distribution systems (e.g., reservoirs, wells, treatment systems, pipelines) and drainage facilities (e.g., bridges, dams, channels, culverts, levees, storm sewers) is designed to provide a sufficient supply of healthy water and to remove physical, chemical, and biological (pathogen) contaminants. When any of these aspects is ignored, water-related disease can emerge; thus, both design and maintenance are crucial to the sustainability of water-related infrastructure.

Much of the water-related infrastructure in developed countries has been designed, built, and operated according to prescriptive codes, standards, regulations, and practices (Neumann et al. 2005). In contrast, infrastructure in developing countries ranges from simple water catchment systems (containers of any sort to harvest and store rain, river, run-off water, etc.) to those rivaling the complexity of those anywhere. Most often, however, much of the water-related infrastructure and management systems in developing countries is grossly deficient because of unreliable and unsustainable water supplies, contamination, inadequate distribution, the high cost of water in some areas, and numerous other reasons (UNEP 2008). Obvious examples of deficient infrastructure include poor drainage or water catchment, which may result in habitats suitable for certain mosquitoes, and inadequate or intermittent pressure/operation of potable water distribution networks, which permits the entry of pathogens and other contaminants. Such factors unquestionably increase the prevalence of infectious disease. From an engineering perspective, innovative although not necessarily complex infrastructure is essential to reduce water-related diseases in poor countries (Mara 2006), especially in urban areas where population density, financial, institutional, and other constraints can preclude elaborate systems. In arid areas, technologies such as dry or low-water-use toilets can help to decouple water supplies from sanitation, thus extending limited water supplies.

Multidisciplinary engineering perspectives have long addressed environmental, economic, social, and institutional elements inherent to large-scale water systems in developed countries. Environmental engineering (formerly called sanitary engineering) addresses, as examples, urbanization and inadequate sanitation that can lead to water pollution; seasonal and annual variability of precipitation and stream flow; climate change that exacerbates water shortages and increases pollution; financial and other resource limitations; and engineering and management expertise (Liu et al. 2007; Medd and Chappells 2007). Economic engineering and risk/cost-benefit analyses recognize the multiple values (e.g., monetary costs and benefits) of water projects, and systems approaches have been used to determine cost-effective designs and operation (e.g., Lund et al. 2006). The natural and social sciences inherently overlap in this work.

### Anthropological approaches

Anthropological studies of water-related disease have focused on several themes including local understandings of water-associated diseases such as dengue fever (Kendall et al. 1991) and diarrheal diseases (Nichter 1988); conceptualizations of water—as pure, unclean, scarce, or having healing properties (Arar 1998; Wellin 1955); water use in treatments such as oral rehydration therapy (ORT) for diarrheal diseases (Burghart 1996); the political economy of health care and access to proper sanitation (Ecks 2004; Obrist 2004); community participation and health education (Yasumaro et al. 1998); and gender, occupational, and cultural inequalities in disease burden (Ramaiah et al. 2000; Vlassoff 1994). Many of these themes are interconnected and overlapping.

Local interpretations and use of water, which appear independent of Western influences, may have important implications for public health. There are many examples: Peruvians with little Western education ignored instructions to boil water and instead preferred “uncooked” water (Wellin 1955); in contrast, many well-educated Sri Lankans also failed to follow boiling instructions (Nichter 1988), whereas women in southern Nepal, when preparing ORT mixes, boiled water according to their own understandings of the effects of heat on water, using traditional procedures for sterilizing milk (Burghart 1996). In Tanzania, among women who accepted public health messages about using clean water, infrastructure deficiencies made it difficult to obtain piped water and created emotional stress on those who wanted but did not have access to it (Obrist 2004); others knew the importance of boiling water, but could not afford the fuel. Thus, water-associated disease risk and prevention is not only economically and politically framed, but often is also socially and behaviorally constrained.

In the absence of adequate and usually state-provided knowledge and resources, social science approaches increasingly recognize adaptive capacity—that is, the ability to cope with change—of individuals, institutions, and even entire social systems. Adaptive capacity considers resilience and, instead of devising interventions that replace old behavior and technology with novel behavior and technology, explores the development of interventions that incrementally alter existing systems (Yacoob and Whiteford 1994). This approach has been offered as a means to improve integrative water management programs in UNESCO’s (United Nations Educational, Scientific and Cultural Organization) Ecohydrology Program (Lemos et al. 2007). Adaptive capacity integrates local knowledge, skills, and traditions at all levels and illustrates that although “disease is a biological condition … it exists within a human and social context” (Yacoob and Whiteford 1995). While admittedly controversial in the sustainability context, adaptation is also a response to climate change.

The anthropological approach also encompasses a shift within medical anthropology from interpretive analyses of the cultural understandings of illness and local classification of diseases to ethnoecologic studies that examine environmental, biological, and social aspects of disease, as well as political/economic approaches in which public health is placed within a larger nexus of power and knowledge (Whiteford and Whiteford 2005). Using interdisciplinary approaches, anthropologists and public health practitioners have worked together to formulate and evaluate appropriate interventions. At the same time, they have examined distal factors, such as national and international aid and loan schemes that contribute to health inequalities, a theme examined next. Rapid ethnographic analyses may prove advantageous in these investigations.

### Economic/political approaches

An essential determinant of water availability and quality is the extent to which political will and economic resources exist among water management agencies (Coit 2002). Further, political approaches must overlap with socio-cultural research to address issues of trust and empowerment. For example, the neglect of sanitary conditions of India’s poor urban areas has been tied to the mistrust of the government: The poor have little or no voice, pay few taxes, and thus are excluded from state-provided resources and services, whereas middle-class residents, who have little confidence in local municipalities to deal with problems, create alternatives (albeit subpar) that do not rely on local governments (Chaplin 1999). Such mistrust is compounded by the culturally sensitive nature of sanitation in India (Rosenquist 2005).

The relevance and validity of indicators and underlying data availability and quality are important concerns. For example, Target 11 of Millennium Development Goal 7 aims to significantly improve lives of 100 million slum dwellers by 2020 (United Nations 2008), as measured, in part, by access to sanitation and the response to the question “Do you have access to a latrine?” WHO (2000) statistics report that 96% of Nairobi’s (Kenya) and 98% of Dar Es Salaam’s (Tanzania) inhabitants have access to sanitation. However, in Nairobi, with half the population in informal settlements, there can be more than 200 people using a single open pit; in Dar, pits frequently overflow in the rainy season and flood the streets with raw sewage (Satterthwaite 2003).

The concentration of poverty implied in the previous discussions of deficient water supply and sanitation infrastructure is a key force behind the spread of water-related (and other) diseases (Massey 2009). Poor people often have greater exposure to pathogens (e.g., crowding may promote disease transmission and vector growth), higher prevalence of underlying diseases, and less access to adequate health services (discussed later). Such social determinants of health, which include both proximal factors (e.g., homelessness, crowding, water supply, and nutrition) and distal factors (employment and development policy), are integrally linked to poverty and act to increase susceptibility to disease.

Another dominant theme for water-related development projects is the influence of donor agendas rather than priorities set by local governments. The structural adjustment policies of the World Bank have deemphasized government-related social projects while promoting market-driven options, for example, privatization of water supply (Stein 2008). Donor project approaches were phased out in the 1990s, and bilateral aid moved toward the pooling of resources to aid economies through sectorwide action programs. However, there are rarely mechanisms (financial, institutional, capacity-related) in place to continue the project when donor contributions end (Rautanen et al. 2006). For example, the termination of the large Swedish International Development Cooperation Agency (SIDA) Health, Sanitation and Water project around Lake Victoria was driven by the decision of SIDA, not the central or local governments of Tanzania, and without consideration of the sustainability of the project (Weeks et al. 2003) The continued donor-coordinated focus on AIDS, tuberculosis, and malaria rather than diarrhea, which is the second leading cause of morbidity and mortality in the country, may also be seen as evidence of a donor-controlled health agenda. Policy spaces need to be generated that empower the historically marginalized and that change the nexus between water, environment, and health. Recent and self-sustaining systems that can help to institutionalize clean water and sanitation habits include microcredit schemes and health-related clubs (Waterkeyn and Cairncross 2005). These economics-driven plans depend on interdisciplinary insights from ecology, engineering, and the social sciences if they are to be technically effective and culturally appropriate.

### Public health approaches

From its beginnings with Snow and Whitehead, public health research has shown the need to address all possible points of water contamination, including sources and storage (Wright et al. 2004), sanitation systems (Cairncross 2003), and hygiene processes (Curtis et al. 2000; Curtis and Cairncross 2003). Public health research areas most germane to water-related infectious disease include surveillance/forecasting, environmental health, epidemiology, interventions, and health education, and the methods to strengthen the relevance and impact of findings in each of these areas. These areas themselves are multidisciplinary and overlapping. Surveillance is critically important and sometimes quite poor, especially in developing countries. For example, although declines in diarrheal mortality have been reported in some settings, true rates may be greater because of reliance on verbal autopsy reports or confounding by other health conditions, such as HIV/AIDS and malaria (Parashar et al. 2003). In addition to health indicators, surveillance can be of water quality and quantity parameters that provide fundamental data for predicting and possibly preventing disease outbreaks by anticipating, for example, impacts of seasonal changes and weather patterns (Fisman 2007).

Many public health interventions have been conducted to evaluate insecticides and vector control techniques (Edman 2005), vaccines (Tetteh and Polley 2007), immunizations, rehydration therapies, and ORT supplements (Bhutta et al. 2000). Such interventions do not always account for socio-cultural traditions and norms and pathogen and human ecology and behavior. Moreover, interventions should be aimed at the incremental reduction of disease rather than eradication (Koren and Crawford-Brown 2004). Another trend is a move beyond risk analysis and risk reduction strategies toward more holistic health-impact assessments, which use ongoing community-oriented evaluations and a broad set of techniques (Patz et al. 2008; Veerman et al. 2005). Such approaches promote the use of preventative measures and early warning systems and can help to close the gap between research and policy (Eisenberg et al. 2002).

Health systems, which are responsible for both curative and preventive care, offer many public health opportunities for provision of clean water and improved sanitation through surveillance, education, and interventions. Yet developing country health posts, clinics, and even referral hospitals themselves suffer from a range of water-related problems. Often, operating and delivery rooms in developing countries dispose of infectious waste in pits and open sewers, and the water supply is neither clean nor reliable. For example, in Uganda, 77% of primary care clinics and 46% of hospitals lacked running water, and only 40% of all facilities had a functioning latrine, an acceptable level of cleanliness, and a protected waiting area (Uganda Service Provision Assessment Survey 2008). Such deficiencies in water and sanitation create opportunities for serious breaches of hygiene that increase the rate of postoperative and other iatrogenic infections.

Despite increasing cross-disciplinary activities, an important disconnect remains between public health research and the social/behavioral and economic/political approaches discussed earlier. Development goals often only indirectly address public health goals (Satterthwaite 2003), while most public health programs notably exclude issues such as political will, economic livelihoods, and resource availability. Still, public health research has led to many successful policies and interventions. The literature shows some agreement about prioritizing interventions; for example, safe and sanitary fecal disposal is of utmost importance; routine hand washing and access to potable drinking water are important, but secondary (Curtis et al. 2000).

### Other interdisciplinary approaches

There have been many earlier efforts to synthesize and integrate disciplinary contributions to water- and health-related research. These include the renowned and ground-breaking science–community–policy nexus (e.g., Brundtland 1997; Lemos et al. 2007), a largely conceptual model useful for recognizing omissions in analyses and motivating interdisciplinarity, but not necessarily one that provides a framework suitable for research and analysis. Another example is integrated water management (IWM), promoted by the World Bank and United Nations and also guided by multidisciplinary (ecologic, institutional, and economic) principles. However, IWM intends to provide a universal solution to very complex problems, rather than a framework for understanding and controlling potential outcomes. It also has been criticized for compartmentalized management and, in the case of flood control, a focus on postdisaster responses rather than predisaster prevention and protection (UNEP 2004). Additional examples of interdisciplinary approaches, mentioned earlier, include WHO’s IVM, UNESCO’s ecohydrology program, and many large-scale research projects emerging from public health.

## Common Problems and Conceptual Needs

Although the disciplinary approaches discussed above have reached out to other disciplines and have provided valuable insight into many aspects of water and health, a systems approach requires more integration. Here, we highlight several common issues associated with water-related research and suggest how interdisciplinary approaches might be used to enhance understanding and improve the effectiveness of interventions.

### Complexity and interactions

The social, ecologic, engineering, economic/political, and public health domains that together determine water and health outcomes are complex, interactive, nonlinear, and dynamic. These processes are difficult to represent using statistical or physically based simulation because of their high-dimensionality, sample-size limitations, which prohibit investigation of most interactions, and unmeasured or unknown spatial and temporal variables (Fleming et al. 2007). Ideally, models should represent a balance between simplicity, which may increase robustness, and complexity, which should enhance realism (Soller and Eisenberg 2008).

### Multiple outcomes

Impacts of water-related projects can be classified using nomenclature from the environmental impact assessment literature: primary impacts directly associated with actions or projects (e.g., presence of pathogens, displacement of communities, channelization) and secondary or indirect impacts (e.g., siltation, ecologic changes, parasite infestation, floods, drought, and community restructuring) that often are much harder to predict yet ultimately more significant. Impacts and causal factors may also be classified by distance as proximal or distal (Birley 1995; Birley and Lock 1999). Clearly, the focus on sustainability has increased the importance of the temporal dimension, for example, short- and long-term (including intergenerational) impacts.

### Unintended consequences

Unintended consequences are a corollary following from these complex interactions and multiple outcomes. Drastic differences may result between short- and long-term outcomes and between local and regional effects. History is rife with examples: flood-control programs that led to flooding or greater prevalence of vector-borne diseases (Saenz et al. 1995; Sur et al. 2000); improperly treated water that irrigated crops and transmitted infectious disease (Liang et al. 2007); and large-scale irrigation projects that promoted mosquito breeding (Amerasinghe and Indrajith 1994; Tyagi and Chaudhary 1997). Additional examples are described in the case study below.

### Data gaps, incompatible temporal and spatial scales of available data, and the cost of environmental monitoring

These items represent additional research challenges. Complete information on all relevant parameters is never available. Rather, information must be gleaned from multiple and sparse data sets, and knowledge and data gaps are common. As examples, linkages among environment, poverty, and health in urban areas are poorly known (Nunan and Satterthwaite 1999), as are complete transmission pathways for all but a few infectious diseases (Patz et al. 2004). Although remote sensing of water quality, water temperature, and soil moisture, as examples, may complement sporadically sampled data, such measurements often are only indirectly related to the main parameters of interest (Brando and Decker 2003; Lavery et al. 1993). Furthermore, the divergent temporal and spatial scales used in sampling may provide incompatible data (Waters Network 2008). Innovative data fusion techniques are needed to optimize the information that can be derived from existing measurements and to support the indicators needed or desired for research, planning, and evaluation.

### Sustainability

Last, the lack of sustainability of water management projects is a daunting problem. Although many health outcomes have been improved in some countries, few interventions in developing countries have had lasting improvements, and most operate only with donor support. Research and policy must understand and appreciate the usefulness of context, which provides information about historical practices and trends in climate, economics, politics, social, and cultural conditions. Although sustainability implies continuity, changes can be rapid and unexpected, and a surprise-rich future should be anticipated. As expressed by Rammel et al. (2007), sustainability can be viewed through an evolutionary lens in which it is understood that while we may not know exactly what will change or how, we can anticipate that change will happen. This demands novel and flexible solutions and systems that can meet evolving circumstances (applying to both environmental and human systems).

## Looking Forward: An Approach for Sustainability Research

A research framework appropriate for creating sustainable solutions to control water-related infectious disease must move beyond existing approaches. We suggest four necessary attributes:

- Interdisciplinary approaches and teaming that account for the complexity, scale, and dynamics of water-related infectious disease problems.
- Ongoing surveillance and monitoring that include not only the traditional public health indicators (such as mortality and morbidity), but also indicators from other relevant disciplines.
- Research agendas that use an extended time horizon on the order of decades—long enough to provide continuity and meaningful progress for data collection, policy development, implementation, and analysis, but short enough to allow system evolution and information updating. Five-year evaluations are suggested below.
- A systems approach that provides an overall framework to facilitate analysis, understand interactions/feedbacks, and promote collaboration among researchers with diverse backgrounds. As discussed below, this framework is well suited to the complex and interdisciplinary nature of the problem.

Overall, the envisioned approach will not only foster responsive cross-disciplinary research collaborations, but will be amenable to on-the-ground implementation, monitoring, and evaluation, as well as the training of a cadre of water and disease specialists and researchers. Note that we propose only a framework for research, not specific programs.

### Systems approach

The systems approach increasingly is considered a useful problem-solving framework to deal with large and complex issues. A system is defined by its interrelated components that function together within a defined and explicit boundary, often to advance a common purpose. Many systems are hierarchical in nature, and some are amenable to computer simulation. Generally, systems methods encompasses iterative steps of defining the problem, gathering data, developing evaluative criteria, formulating and evaluating alternatives, and selecting, designing, and implementing the plan (Jewell 1986). Many disciplines contribute to these steps. By acknowledging the relationships among system components, specifically the feedback and reversibility of many interactions, the approach allows evaluation of alternative courses of action or scenarios. The systems approach strives for straightforward problem definition, assumptions, goals, objectives, and evaluative criteria, and it allows continuous assessment and updating as new information becomes available. Another strength is its ability to facilitate problem mitigation and active planning by identifying the processes and parameters that influence key outcomes (called sensitivity analysis) (Bender and Simonovic 2000; Simonovic 2000). Many water management problems have applied a systems approach using computer models to simulate physical processes (Jeppsson and Hellstrom 2002; Lund et al. 2006) and institutional factors, such as the capacity for interagency and organizational collaboration (Cassady et al. 2008; Temel 2005).

**Figure 2:**
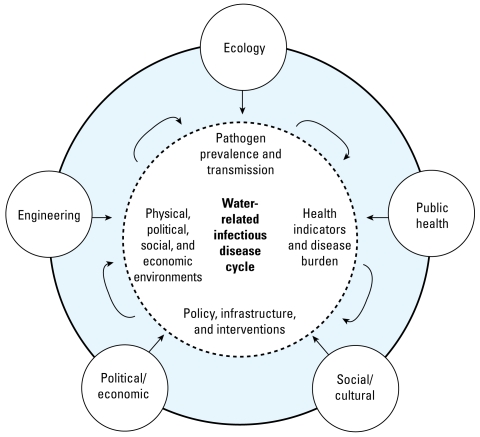
Conceptual framework of multidisciplinary health-based systems approach for understanding the water-related infectious disease cycle.

The proposed research framework acknowledges the dynamics of infectious disease transmission and integrates the disciplinary approaches. Figure 2 depicts linkages of key processes within the water-related infectious disease cycle. The four key interrelated components that constitute the water-related infectious disease transmission cycle are represented near the center of the figure:

- Pathogen prevalence and transmission correspond to vector ecology and proximal factors that affect vector breeding and pathogen transmission, for example, the presence of standing water.
- Relevant health indicators and disease bur-den represent results from surveillance and monitoring that show the status and trends of both ecologic and human health. Such indicators are selected and developed using knowledge of disease ecology within the context of existing surveillance systems for environmental and public health and can include standard measures, such as mortality, morbidity, and infection rates, and those tailored to local circumstances.
- Policy, infrastructure and interventions represent actions designed to influence human behavior in a positive manner, reduce risks of transmission, and otherwise lessen the disease burden, for example, improved hygiene and water safety.
- All decisions and interventions are made within the context and constraints of physical, political, economic, and social environments, for example, cultural views of water and hygiene, and the available economic resources.

In Figure 2, we highlight the interdisciplinary teaming within a systems framework, specifically the five frames reviewed above (outer circles), designed to capture the complex dynamics and multiple temporal and spatial scales of water-related infectious disease problems. Rather than reducing the validity of traditional approaches, the proposed framework is intended to be integrative and problem oriented, incorporating these and other relevant and helpful disciplines or techniques. Although a systems approach is, by definition, multidisciplinary, we are calling for a deeper integration and collaboration between scientists in which constituent disciplines inform investigations of others and where hypotheses might even be jointly formed (Wear 1999).

The proposed approach is driven by the need to better address the burden of water-associated diseases, with improved human health being the principal objective. It could be argued that providing adequate access to water and proper sanitation and possibly fulfilling the needs and/or desires of communities are sufficient and more appropriate goals. As described above, however, most characteristics of the water-related infectious disease cycle (e.g., complexity, unintended consequences, data incompatibility, long time horizon, and interplay within the social/cultural/economic and other frames) suggest that health indicators are more relevant and more consistent than economic, political, or infrastructural indicators, which tend to be less stable, more susceptible to change, and sometimes irrelevant under changed circumstances. Furthermore, many health indicators are becoming increasingly standardized in terms of their definitions and data collection methods (Centers for Disease Control and Prevention 2006). For example, in the context of sanitary and water supply improvements, the focus would not be on the level of expenditures on new sewage systems, but on levels and trends of pathogens in the water supply, incidence trends of diarrhea, the numbers of outpatient visits to clinics for dysentery and intestinal worms, and the like. The proposed framework explicitly shifts the focus to health-based goals rather than economic targets and thus represents a radical reorientation for most development programs. Some movement in this direction is evidenced by a U.S. Environmental Protection Agency grant opportunity (Pongsiri and Roman 2007), a World Bank (2008) review that emphasized collaborative research (especially in exploring linkages between environmental sustainability and poverty), and recent discussions in the National Science Foundation/National Institutes of Health collaborative program on the ecology of infectious disease (National Institutes of Health 1999).

We also suggest that the research framework can be embedded in the policy process, following the approach taken in health impact assessment, discussed earlier—a structure that might help provide continuity of support. This may also be valuable for young and interdisciplinary research investigators, as officially sanctioned projects might help surmount some of the challenges of establishing trust and understanding among members of the research team, as well as help in funding and publishing interdisciplinary research (Turner and Carpenter 1999).

Ongoing evaluation is a critical component of the proposed approach. At the project level, evaluations must address environmental quality, impacts on livelihoods, health, household economies, and the overall cost and acceptability of the action. Distributional effects need to be carefully assessed to ensure equity of benefits. At a programmatic level, interventions and policy experiments should be evaluated for their intellectual contributions, repeatability, fostering of effective collaboration, and training opportunities, among others. Given the 20-year time horizon we argue for, formal evaluations might be conducted every 5 years by interdisciplinary researchers, a schedule allowing for evolution and the opportunity to modify components within the entire program as well as promoting the training of new investigators. Up-front structuring of data collection activities and indicators obviously would facilitate evaluation.

### Complementary research tools

The proposed research framework needs to incorporate the latest research tools, including geographic information systems (GIS), process and simulation models, and statistical techniques. GIS has been used for many purposes, including designing early-warning systems (Fleming et al. 2007) and tracking disease outbreaks (Sarkar et al. 2007). Combined with satellite surveillance, it has been used to map vector breeding sites and other disease sources (Jacob et al. 2003; Njemanze et al. 1999; Polack et al. 2005). A wide range of models are used to study environmental changes and impacts on health and sometimes combined to study dynamics and interactions among environmental, ecologic, social, and pathogen factors that affect disease transmission (Eisenberg et al. 2007; Remais et al. 2008).

Given the complexity of water-associated infectious disease, statistical data mining and variable selection techniques using tree-based searches through the model space (Breiman 2001) may be useful. Latent variable models under a structural equation framework may provide an option for understanding causal pathways and interacting factors that lead to disease transmission. The study of water-related diseases inevitably involves various spatiotemporal covariates, with the spatial variables themselves often measured at different scales and with a nested interface. Such problems can be analyzed using wavelet-based methods, spatial process models, hierarchical or multilevel modeling frameworks, and Bayesian inferential methods (Banerjee et al. 2004), although the application of these methods to date has been limited. There are many opportunities to use these tools to great advantage in the evidence-based decision-making public health paradigm.

## Case Study

A case study of water-associated disease in Ecuador illustrates the need for and application of the proposed approach. With the goal of providing transportation faster and cheaper than river boats, the Ecuadorian government built a 100-km road between the southern Colombian border and the Ecuadorian coast from 1996 to 2001. After completion of the main road, secondary roads continued to be built that linked multiple villages to the main road. This roadway network led to major changes in both the social structure and ecology of the region (Sierra 1999). Although there is evidence that road construction affects the incidence of vector-borne and sexually transmitted diseases (Birley 1995), impacts on diarrheal disease remain poorly understood. Further, although transmission of enteric pathogens has been linked to proximal factors of water quality, sanitation and hygiene practices, the relationship between distal social and ecologic factors (e.g., increased population density and regional scale water patterns) and diarrheal disease remains poorly understood (Curriero et al. 2001).

**Figure 3:**
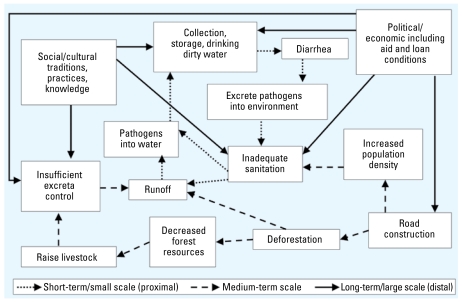
Example of distal, medium-term, and proximal components in the water-related infectious disease cycle in the Ecuadorian case study.

To help understand road construction related diarrheal disease, Eisenberg et al. (2007) mapped a suite of distal environmental changes that can affect proximal environmental characteristics, which in turn can affect the transmission of enteric pathogens. Their framework incorporates processes at multiple spatial and temporal scales using regional, villagewide, individual, and molecular-level data. These data can be integrated using systems approach. To demonstrate the system’s complexity, multiple outcomes, and the potential for unanticipated consequences, consider how road construction can affect diarrheal disease prevalence: Roads can lead to deforestation, which subsequently affects watershed hydrology, local climate, and pathogen transmission (Figure 3) (Curriero et al. 2001). Roads also increase flows of consumer products, material goods, and medicine and potentially improve access to health care facilities and health information. At the same time, short-term travel patterns are intensified, introducing pathogen strains into the communities. The population density in both existing and new communities created along the new roads can rapidly increase, but water supply and sanitation infrastructure frequently lags, thus increasing the likelihood of transmission of enteric pathogens. Similar changes can also be produced by dams, urbanization, agricultural practices, deforestation, and climate change.

Interventions at multiple points in the cycle and at different temporal and spatial scales can break the pathogen transmission cycle. For example, Figure 3 shows that although road construction and the resulting deforestation are linked to disease transmission, these medium-scale drivers are ultimately linked to distal drivers, such as country-level economic conditions and the agendas of development aid/loan programs. Deforestation can then lead to proximal drivers—such as soil runoff that increases pathogen transmission into water sources, and decreased forest resources—potentially leading to more intensive livestock husbandry to compensate for lost forest resources, which then may increase the risk of pathogen transmission via inadequate control of waste. An interdisciplinary systems approach can account for the varying temporal and spatial scales depicted in the figure and will foster collaboration in the collection and evaluation of data. Table 1 outlines several measurable indicators relevant to health and sustainability within the water-related infectious disease cycle appropriate for the Ecuadorian example and identifies key contributing disciplines. The table highlights the need for interdisciplinary collaboration starting with conceptualization of the approach and continuing throughout.

This case study demonstrates the significant and intersecting roles played by multiple disciplines in understanding the causal linkages between road construction and disease. As examples, the political/economic disciplines shed light on why the road was built and its impact on the local economy of the region, whereas anthropology/ethnoecology studies describe the relationships between these larger-scale political/economic factors and the community’s social structures and how these affect behaviors, services, and infrastructures needed for disease prevention. In an analogous fashion, ecologic sciences and engineering describe impacts of the larger-scale processes on the environment in general and ultimately on water quality, and they also offer input into the assessment and design of actions to mitigate adverse environmental (and health) impacts. Public health has the role of both measuring the occurrence of disease through surveillance activities and evaluating the effectiveness of possible interventions. The challenge in such studies is to make these activities truly integrated and interdisciplinary. Barriers include differences in terminology and theoretical frameworks, which require working together to create protocols for collecting and analyzing data, and the need for sustained financial and institutional support, which can develop local capacity, understand the complex relationships, and ideally move beyond observational studies into intervention research. Ecuador could become a test case that both demonstrates the value of the proposed research approach and leads to improved health. Although many of the linkages between road construction and disease may be case-specific, such studies would show the utility of an interdisciplinary systems approach framework that incorporates the dynamics of infectious disease transmission within the social, ecologic, engineering, economic/political, and public health spheres discussed in this review.

## Conclusions

We have argued that fundamental changes are needed in the structure and organization of research on water-related infectious disease and specifically for a systems- and health-based approach that can lead to sustainable strategies. After reviewing contributions of the key disciplines, we highlighted important themes and conceptual needs that include the complexity of and linkages (both proximal and distal) across ecologic, engineering, political, economic, anthropological, and public health spheres; the need to integrate data and methods used in the relevant disciplines, including surveillance activities tracking public health and other short-term and long-term indicators; and the multiple and often unanticipated outcomes as well as the long time frame needed to consider the sustainability of interventions addressing water-related diseases, all of which motivate an adaptable research framework. A research agenda using an interdisciplinary systems framework with an evidence-informed health outcomes focus and extended time horizon is responsive to these issues and builds on recent trends, although it may diverge from goals of the (economic) development paradigm. We used many short examples and a case study to illustrate the dynamics and complexity inherent in these problems. These show the ripe opportunities for interdisciplinary collaboration in data collection, analysis, and evaluation. The suggested systems-based research framework is amenable to methods and data culled from the ecologic, anthropological, and engineering fields, among others, and it facilitates knowledge sharing across the diverse disciplines involved. It can be embedded, we believe, in new initiatives in educational curriculum, research programs, policies, and intervention programs designed to control water-related infectious disease. This review is an initial step toward these goals.

Many challenges remain. More time and flexibility may be needed than is customary in disciplinary research. Interdisciplinary training at theoretical, methodologic, and analytical levels is needed. Program priorities and funding opportunities must be shifted. There has never been a more pressing yet more propitious time for such changes in approach, shifts in paradigms, and development of new interdisciplinary collaborations.

## Figures and Tables

**Table 1:** Examples of indicators for water-related disease and control and the primary disciplines needed to contribute in the formulation and evaluation of each indicator.

Indicator	Social/cultural	Political/economic	Ecology	Health	Engineering
Changes in water quality and conditions (flow frequency, intensity, path; pollution and pathogen levels)			X	X	X

Ecologic and infrastructural capacity to withstand climatic changes (temperature fluctuations, rainfall variability)			X		X

Household- and community-level motivation for changes in water-seeking and water-using behaviors; intervention acceptability	X			X	

Human migration, settlement, and water-use patterns	X	X		X	

Economic conditions, access to potable water and health care providers		X		X	

Multiple disciplines contribute to most indicators.
